# The relationship between vegetable intake and chronic gastric disorder among community-dwelling residents aged 35 years and older in China

**DOI:** 10.3389/fnut.2025.1696598

**Published:** 2025-10-29

**Authors:** Yue Guan, Yu Dong, Huiqing Xu, Yunting Xu, Guofeng Ao, Jing Ji, Jie Yuan, Yan Zhang, Qing Ye

**Affiliations:** ^1^Nanjing First Hospital, Nanjing Medical University, Nanjing, China; ^2^Nanjing Medical University Affiliated Nanjing Municipal Center for Disease Control and Prevention, Nanjing, China

**Keywords:** chronic atrophic gastritis, chronic gastric ulcer, chronic gastric disorder, vegetable intake, Chinese adult

## Abstract

**Background:**

Chronic gastric disorder (CGD) is a common disease among people in China. However, very few studies are available on the association between lifestyle factors, such as vegetable consumption, and CGD. This study aimed to investigate the relationship between vegetable intake and CGD among adults in regional China.

**Methods:**

In this cross-sectional survey conducted in 2023, a total of 38,051 community-dwelling residents aged 35 years and older were randomly selected from the Nanjing municipality in China. The outcome event was self-reported CGD, referring to chronic atrophic gastritis (CAG) or chronic gastric ulcer (CGU). The vegetable intake level was assessed using a validated food frequency questionnaire (FFQ) and classified into two subgroups based on the consumption recommendation (≥300 g/day), as advised by the Chinese Nutrition Society (CNS) and tertiles. Logistic regression analyses were performed to compute the odds ratio (OR) and 95% confidence interval (CI) in order to examine the associations between vegetable intake and CGD.

**Results:**

Among the participants analyzed, the overall prevalence of CGD was 16.6%. Moreover, the proportion of participants who met the vegetable consumption recommendation was 32.0%. After adjusting for potential confounding factors, participants who met the recommended vegetable intake had significantly higher odds of experiencing CGD (OR = 1.16, 95%CI = 1.10, 1.23) compared to those who did not meet the recommendation. Additionally, adults in the high (OR = 1.26, 95%CI = 1.18, 1.35) and middle (OR = 1.16, 95%CI = 1.09, 1.24) tertiles of vegetable intake were also more likely to experience CGD compared to those in the low tertile. Furthermore, the positive associations between vegetable intake (defined by either the recommended level or tertiles) and CGD were consistently observed across subgroups stratified by age, sex, or residential area.

**Conclusion:**

Vegetable intake was found to be positively associated with CGD among community-dwelling residents aged 35 years and older, both overall and across subgroups stratified by age, sex, or residential location in regional China. This study suggests that vegetable intake may be an influencing factor in the prevention of CGD among adults in China. The study also indicates that population-level interventions promoting vegetable consumption could be a viable option for preventing CGD.

## Introduction

Chronic atrophic gastritis (CAG) and chronic gastric ulcer (CGU), two common types of chronic gastric disorder (CGD), are known risk factors for gastric cancer (GC) (1). The prevalence of GC has continued to rise over the past decades in China, although its incidence rate has gradually declined (2). Currently, GC is the second most common cancer and the second leading cause of cancer-related deaths in China (3). Considering that CGD is an influencing factor of GC, it is reasonable to infer that the burden of GC could be reduced by implementing community-based prevention strategies for CGD in China. Therefore, it is imperative to identify the modifiable risk factors associated with CGD and subsequently implement tailored interventions at the population level to address these factors effectively.

It is estimated that the average prevalence of CGD is >20% among adults in China, which not only contributes substantially to the disease burden itself but also indicates a potential burden of GC (4). Vegetable intake, a modifiable lifestyle factor, is crucial for human health and has been recently examined for its potential relationship with CGD (5–8). However, the conclusions from these studies have not been consistent, showing a negative or null association between vegetable intake and the risk of CGD (5–8). Particularly, among Chinese adults, one study reported a negative association between vegetable intake and CGD (6), whereas another study documented a null relationship between them (8). This highlights that further investigation should be conducted to examine the relationship between vegetable intake and CGD among representative populations in China.

Due to the high prevalence of both CGD and GC in China, and the inconclusive findings regarding the relationship between vegetable intake and CGD, further studies are needed to provide additional evidence on this association. Therefore, we conducted a study specifically to explore the association between vegetable intake and CGD among representative community-dwelling adults in regional China, both overall and across subgroups stratified by age, sex, and residential area.

## Methods

### Study design and participants

The study was conducted in the Nanjing municipality, a megacity in eastern China. It had approximately 9.3 million registered residents and 12 (five urban and seven suburban) districts in 2020, which is the most recent census year (9, 10). This cross-sectional study conducted in 2023 primarily aimed to investigate common chronic non-communicable diseases (NCDs) and the associated risk factors. The common NCDs mainly included gastric disorder (chronic gastritis or stomach ulcer), hypertension (HTN), and diabetes, while NCD-related risk factors comprised vegetable and meat consumption, smoking, drinking, physical activity (PA), sedentary behavior (SB), body weight status, and lipid profile.

The inclusion criteria for participants were as follows: (1) community-dwelling residents registered in the study districts, (2) age: 35 years and older, (3) no cognitive/psychiatric disorders, (4) not experiencing acute infectious disease, and (5) no history of cancer. For sample size estimation, we aimed to ensure that sufficient outcome events could be identified within each participating district and to warrant that the association between vegetable intake and the outcome event could be examined with sufficient statistical power in each district. Therefore, the study sample size was first calculated at the district level. Based on the following factors: (1) an average CGD prevalence of 20% among adults in China (4); (2) the cross-sectional study design; (3) a multi-stage cluster sampling approach; (4) an expected response rate (85%) and statistical power (90%); and (5) stratified analysis by age, sex, and residential area, it was estimated that the district-level sample size was approximately 6,000. Then, considering that five districts were included in the entire study, the overall sample size was approximately 30,000 at the municipality level.

Participants were randomly recruited using a multi-stage sampling approach. First, considering that there were five urban and seven suburban districts in Nanjing in 2023, to maximize the representativeness of participants, two urban and three suburban districts were randomly selected from all the 12 districts. Next, all administrative streets and townships within the five selected districts were included in the study. Then, in each administrative street or township, four communities or villages were randomly selected. Subsequently, two neighborhoods were selected from each identified administrative community or village. Finally, a minimum of 60 households were randomly chosen from each involved neighborhood, and all household members aged 35 years and older were potential participants. Consequently, 42,185 residents were eligible to participate in the study based on the assumption that each household typically had two members aged 35 years and older.

Each participant provided written consent. The original experiment was reviewed and approved by the Ethics Committee of Nanjing Medical University Affiliated Nanjing Municipal Center for Disease Prevention and Control. The study methods were developed in line with the principles of the Declaration of Helsinki. As only de-identified data were analyzed in the present study, ethical approval was waived by the Ethics Committee of Nanjing First Hospital, Nanjing Medical University.

### Data collection

In 2018, the Chinese Center for Disease Control and Prevention (CCDC) released the “Scheme of the Chinese chronic non-communicable disease and risk factor surveillance” for the national surveillance of NCDs and their risk factors in China (11). In this scheme, the CCDC recommended core survey contents, questionnaires and instruments, anthropometric measurements, laboratory examination items, and standardized investigation procedures for NCD-related epidemiological studies in China (11). The participants were invited to the local community health service center for an interview on an appointed date (11). The information collected, questionnaires and instruments employed, and anthropometric measures in this study were adopted from the scheme recommended by the CCDC (11).

### Study variables

#### Outcome variable

The outcome measure was self-reported CGD, referring to CAG or CGU in this study. In China, hospitals are classified into grades 1, 2, or 3, with higher grades indicating a higher level of care. Only gastroenterology physicians registered at grade 2/3 hospitals are qualified to make a diagnosis of CGD for patients based on endoscopic and/or histologic examinations (12, 13). In this study, the participants were asked to respond to the following question: Were you ever diagnosed with a chronic gastric disorder (specifically, chronic atrophic gastritis or chronic gastric ulcer) by a registered gastroenterological physician? The response option was “Yes” or “No” (11). Then, the participants who answered “Yes” to this question item were defined as having a positive event. For analysis, the participants were categorized as “having CGD” or “no CGD.”

#### Independent variable

The independent variable was vegetable intake, which was measured using the validated Chinese version of the food frequency questionnaire (FFQ) (14). Two sequential question items in the FFQ were used to assess vegetable intake (14). Item 1 was “How often did you consume vegetable under a typical situation in last year?” [sic] The response option was “Please specify the consumption frequencies” (14). The participants who specified an intake frequency of ≥1 per month were then asked to respond to Item 2: “On average, how many of vegetable did you consume each time?” [sic] The answer option was “Please specify the amount in LIANG (50 g) of vegetable you consumed each time” (14). [sic] The total amount of vegetable consumed per day was calculated for each participant in the analysis.

In 2022, the Chinese Nutrition Society (CNS) released updated dietary recommendations for Chinese residents (15). A minimum vegetable intake of 300 g every day was recommended for Chinese adults (15). Therefore, to examine the relationship between vegetable intake and CGD, the participants were classified as “consumed less vegetables (<300 g/d)” or “consumed adequate vegetables (≥300 g/d)” in this study (15). Moreover, to further analyze its association with CGD, vegetable intake was also categorized into three subgroups: low, middle, and high tertiles (cutoffs: 150 and 250 g/day).

### Covariates

Established risk factors for CGD were adjusted for in the study. Key sociodemographic variables included the following: age (35–59 or 60 + years old), sex (male or female), location (urban or suburban), educational level (≤9, 10–12, or ≥13 years of schooling), and marital status (single or married/having a partner). Lifestyle and behavioral factors included smoking, drinking, PA, and SB. The participants were classified as “smokers” or “non-smokers” and “drinkers” or “non-drinkers” based on the definitions recommended by the CCDC (11). Red meat and white meat consumption were separately measured using the FFQ (14). The CNS does not differentiate between red and white meat in its dietary recommendations for Chinese adults but suggests that an adult should consume 300-500 g of meat per week (15). Therefore, red and white meat intake was combined to categorize the participants into three groups: “consumed less meat (<300 g/wk),” “consumed adequate meat (300-500 g/wk),” or “consumed too much meat (≥500 g/wk) (15).”

PA and SB were measured using the Chinese version of the International Physical Activity Questionnaire (IPAQ-CHN) (16). Moderate and vigorous PA time during the past week was assessed separately. Then, the sum of weekly moderate PA time plus twice the vigorous PA time was calculated to categorize the participants as having “insufficient PA (<150 min/wk)” or “sufficient PA (≥150 min/wk)” (17). SB was measured by the average daily time spent on screen-viewing and used to classify the participants as having “prolonged SB (≥2 h/day)” or “shortened SB (<2 h/day)” based on the specific recommendations for Chinese adults (17).

The participants self-reported their personal histories of HTN, diabetes, and lipid profiles (including cholesterol, triglycerides, and high−/low-density lipoprotein levels), as well as parental family histories of HTN and diabetes. The definitions of these common NCDs and family histories were adopted from the recommendations by the CCDC (11). Then, they were categorized as “hypertensive patient” or “normotensive individual” and “diabetic patient” or “non-diabetic individual” (11). In addition, the participants were classified as “having a normal lipid profile” only when all self-reported levels of cholesterol, triglycerides, and high−/low-density lipoproteins were normal; otherwise, they were classified as “having an abnormal lipid profile” (11). Moreover, the family history of hypertension and diabetes was categorized into “positive (father/mother having the disease)” or “negative (none of the parents have the disease)” separately (11).

For anthropometric measurements, body weight was measured twice to the nearest 0.1 kg, and body height was also measured twice to the nearest 0.01 meter (11). The mean values of body weight and height were used to compute body mass index (BMI) as weight (kg) divided by height squared (m^2^). The participants were classified into four BMI categories: “BMI < 18.5 (underweight),” “18.5 ≤ BMI < 24.0 (normal weight),” “24.0 ≤ BMI<28.0 (overweight),” or “BMI ≥ 28.0 (obesity)” in the analysis according to the recommendations for Chinese adults (18).

### Data analysis

First, descriptive analysis was conducted to present the distribution of the selected participants’ characteristics and CGD prevalence (%). The difference in CGD prevalence across participant attributes was examined using a chi-squared test. Then, logistic regression models were used to estimate the odds ratio (OR) and 95% confidence interval (95% CI) to examine the relationship between vegetable intake and CGD. Model 1 included vegetable consumption as the independent variable with control for community-level clustering effects. Model 2 was a multivariate analysis with adjustment for participants’ sociodemographic attributes (age [where applicable], sex [where applicable], residential location [where applicable], education, and marital status), BMI category, PA, SB, meat consumption, smoking, drinking, hypertension, diabetes, lipid profile status, and family histories of hypertension and diabetes, in addition to the variable included in Model 1. A *p*-value of < 0.05 (two-sided) was considered statistically significant. The data were analyzed using SPSS 23.0 (SPSS Inc., Chicago, IL, USA).

## Results

In total, 42,185 participants were selected. Among them, 38,051 (response rate = 90.2%) responded to the study. “Not available” and “refuse” were the reasons for those who did not respond to the survey. There was no significant difference in age and sex between those who responded and those who did not respond. Table 1 shows the selected participants’ characteristics by area. Overall, 38.9% of the analyzed participants lived in urban areas, while 61.1% resided in suburban areas. Moreover, 31.1% of the participants were adults aged 60 years and older, while 48.5% were male individuals. In addition, 22.4% of the participants had attained college-level education, and only 6.3% were single. Very few participants (1.6%) were underweight, while 42.0 and 18.4% of the participants were overweight and obese, respectively.

**Table 1:** Selected characteristics of the participants by residential location in Nanjing municipality, China, 2023 (*N* = 38,051).

Study design and participants	Residential location, % (*n*)		
	Overall	Urban	Suburban
Total	38,051	38.9 (14800)	61.1 (23251)
Age (years)			
35–59	58.9 (26200)	67.5 (9984)	69.7 (16216)
60+	31.1 (11851)	32.5 (4816)	30.3 (7035)
Sex			
Male	48.5 (18469)	49.0 (7253)	48.2 (11216)
Female	51.5 (19582)	51.0 (7547)	51.8 (12035)
Education (years of schooling)			
9	54.9 (20897)	42.0 (6214)	63.1 (14683)
10–12	22.7 (8624)	26.6 (3934)	20.2 (4690)
13+	22.4 (8530)	31.4 (4652)	16.7 (3878)
Marital status			
Single	6.3 (2403)	6.5 (962)	6.2 (1441)
Married/having a partner	93.7 (35648)	93.5 (13838)	93.8 (21810)
Body weight status^$^			
Underweight	1.6 (623)	1.8 (270)	1.5 (353)
Normal	38.0 (14462)	41.1 (6080)	36.1 (8382)
Overweight	42.0 (15979)	40.8 (6031)	42.8 (9948)
Obese	18.4 (6987)	16.3 (2419)	19.6 (4568)
Smoking^#^			
No	74.9 (28483)	79.7 (11795)	71.8 (16688)
Yes	25.1 (9568)	20.3 (3005)	28.2 (6563)
Drinking^†^			
No	72.2 (27475)	75.4 (11163)	70.2 (16312)
Yes	27.8 (10576)	24.6 (3637)	29.8 (6939)
Physical activity^‡^			
Insufficient	82.3 (31326)	82.5 (12205)	82.2 (19121)
Sufficient	17.7 (6725)	17.5 (2595)	17.8 (4130)
Meat consumption^¶^			
Less than recommended	16.1 (6112)	13.1 (1937)	18.0 (4175)
Within the recommended range	20.3 (7731)	18.5 (2734)	21.5 (4997)
Exceeds recommendation	63.6 (24208)	68.4 (10129)	60.5 (14079)

$ Body weight status was categorized as underweight (BMI<18.5), normal weight (BMI = 18.5, 23.9), overweight (BMI = 24.0, 27.9), and obese (BMI ≥28.0) based on recommendations for Chinese adults using body mass index (BMI).

# Participants were classified as “smokers” or “non-smokers” based on definitions recommended by the Chinese Center for Disease Control and Prevention.

† Participants were classified as “drinkers” or “non-drinkers” based on definitions recommended by the Chinese Center for Disease Control and Prevention.

‡ The sum of weekly moderate PA time plus twice the vigorous PA time was calculated to categorize the participants as having “insufficient PA (<150 min/wk)” or “sufficient PA (≥150 min/wk)”.

¶ Participants were categorized into “less than recommended (<300 g/wk),” “within the recommended range (300-500 g/wk),” and “exceeds recommendation (≥500 g/wk) (15)”.

Table 2 presents the distribution of the participants by vegetable intake recommendation and CGDs across selected characteristics. Among all participants, only 32.0% (95%CI = 31.5, 32.5) consumed vegetables at the recommended level. The older adults (aged 60 + years *vs.* aged 35–59 years: 33.1% *vs.* 31.5%) and suburban residents (suburban *vs.* urban: 35.1% *vs.* 27.1%) tended to consume vegetables at the recommended level. In contrast, the proportion of participants who met the vegetable intake recommendation did not differ by sex (male *vs.* female: 31.9% *vs.* 32.1%) or marital status (single *vs.* having a partner: 30.8% *vs.* 32.1%). Interestingly, the proportion of participants who met the vegetable intake recommendation was lower among those with a higher educational level (13 + years of schooling *vs.* 9 years: 26.0% *vs.* 34.4%) and those who were underweight (underweight *vs.* obesity: 29.2% *vs.* 32.8%) compared to their counterparts with lower education or obesity, respectively.

**Table 2:** Distribution of the consumption recommendation of vegetables and chronic gastric disorder by selected characteristics in Nanjing municipality, China, 2023 (*N* = 38,051).

Study design and participants	% (*n*) of participants				
	Total	Recommendation of vegetable consumption^*^		Status of chronic gastric disorder^**^	
		< 300 g/d	≥ 300 g/d	Without chronic gastric disorder	With chronic gastric disorder
Overall	38,051	68.0 (25874)	32.0 (12177)	83.4 (31742)	16.6 (6309)
Age (years)					
35–59	58.9 (26200)	69.4 (17947)	67.8 (8253)	69.4 (22042)	65.9 (4158)
60+	31.1 (11851)	30.6 (7927)	32.2 (3924)	30.6 (9700)	34.1 (2151)
Sex					
Male	48.5 (18469)	48.6 (12583)	48.3 (5886)	50.0 (15866)	41.3 (2603)
Female	51.5 (19582)	51.4 (13291)	51.7 (6291)	50.0 (15876)	58.7 (3706)
Location					
Urban	38.9 (14800)	41.7 (10783)	33.0 (4017)	40.6 (12882)	30.4 (1918)
Suburban	61.1 (23251)	58.3 (15091)	67.0 (8160)	59.4 (18860)	60.6 (4391)
Educational attainment (years of schooling)					
9	54.9 (20897)	53.0 (13702)	59.1 (7195)	54.4 (17279)	57.3 (3618)
10–12	22.7 (8624)	22.6 (5857)	22.7 (2767)	22.7 (7199)	22.6 (1425)
13+	22.4 (8530)	24.4 (6315)	18.2 (2215)	22.9 (7264)	20.1 (1266)
Marital status					
Single	6.3 (2403)	6.4 (1664)	6.1 (739)	6.2 (1965)	6.9 (438)
Married/having a partner	93.7 (35648)	93.6 (24210)	93.9 (11438)	93.8 (29777)	93.1 (5871)
Body weight status^$^					
Underweight	1.6 (623)	1.7 (441)	1.5 (182)	1.5 (483)	2.2 (140)
Normal	38.0 (14462)	38.5 (9972)	36.9 (4490)	37.3 (11829)	41.7 (2633)
Overweight	42.0 (15979)	41.7 (10768)	42.8 (5211)	42.3 (13443)	40.2 (2536)
Obese	18.4 (6987)	18.1 (4693)	18.8 (2294)	18.9 (5987)	15.9 (1000)
Smoking^#^					
No	74.9 (28483)	75.9 (19632)	72.7 (8851)	74.6 (23694)	75.9 (4789)
Yes	25.1 (9568)	24.1 (6242)	27.3 (3326)	25.4 (8048)	24.1 (1520)
Drinking^†^					
No	72.2 (27475)	73.0 (18888)	70.5 (8587)	72.1 (22888)	72.7 (4587)
Yes	27.8 (10576)	27.0 (6986)	29.5 (3590)	27.9 (8854)	27.3 (1722)
Physical activity (PA)^‡^					
Insufficient	82.3 (31326)	83.5 (21617)	79.7 (9709)	82.8 (26296)	79.7 (5030)
Sufficient	17.7 (6725)	16.5 (4257)	20.3 (2468)	17.2 (5446)	20.3 (1279)
Meat consumption^¶^					
Less than recommended	16.1 (6112)	17.4 (4504)	13.2 (1608)	15.7 (4990)	17.8 (1122)
Within the recommended range	20.3 (7731)	20.9 (5411)	19.1 (2320)	20.2 (6396)	21.1 (1335)
Exceeds recommendation	63.6 (24208)	61.7 (15959)	67.7 (8249)	64.1 (20356)	61.1 (3852)

* Vegetable consumption recommendation: consumption of ≥300 g/d of fresh vegetables was recommended for every Chinese adult by the Chinese Nutrition Society (CNS) in 2022.

**Gastric disorder refers to chronic gastritis or gastric ulcer.

$ Body weight status was categorized as underweight (BMI<18.5), normal weight (BMI = 18.5, 23.9), overweight (BMI = 24.0, 27.9), and obese (BMI ≥28.0) based on recommendations for Chinese adults using body mass index (BMI).

# Participants were classified as “smokers” or “non-smokers” based on definitions recommended by the Chinese Center for Disease Control and Prevention.

† Participants were classified as “drinkers” or “non-drinkers” based on definitions recommended by the Chinese Center for Disease Control and Prevention.

‡ The sum of weekly moderate PA time plus twice the vigorous PA time was calculated to categorize the participants as having “insufficient PA (<150 min/wk)” or “sufficient PA (≥150 min/wk)”.

¶ Participants were categorized into “less than recommended (<300 g/wk),” “within the recommended range (300-500 g/wk),” and “exceeds recommendation (≥500 g/wk) (15)”.

The overall prevalence of CGD was 16.6% (95%CI = 16.2, 17.0) among the participants aged 35 years and older in regional China. Older adults (aged 60 + years *vs.* aged 35–59 years: 18.2% *vs.* 15.9%), female individuals (female *vs.* male: 18.9% *vs.* 14.1%), suburban residents (suburban *vs.* urban: 18.9% *vs.* 13.0%), and single participants (single *vs.* having a partner: 18.2% *vs.* 16.5%) were more likely to experience CGD (*p* < 0.05). Moreover, CGD prevalence was significantly lower in the adults with a higher educational level (13 + years of schooling *vs.* 9 years: 14.8% *vs.* 17.3%) or obesity (obesity *vs.* underweight: 14.3% *vs.* 22.5%) compared to their counterparts, separately (*p* < 0.05).

**Figure 1:**
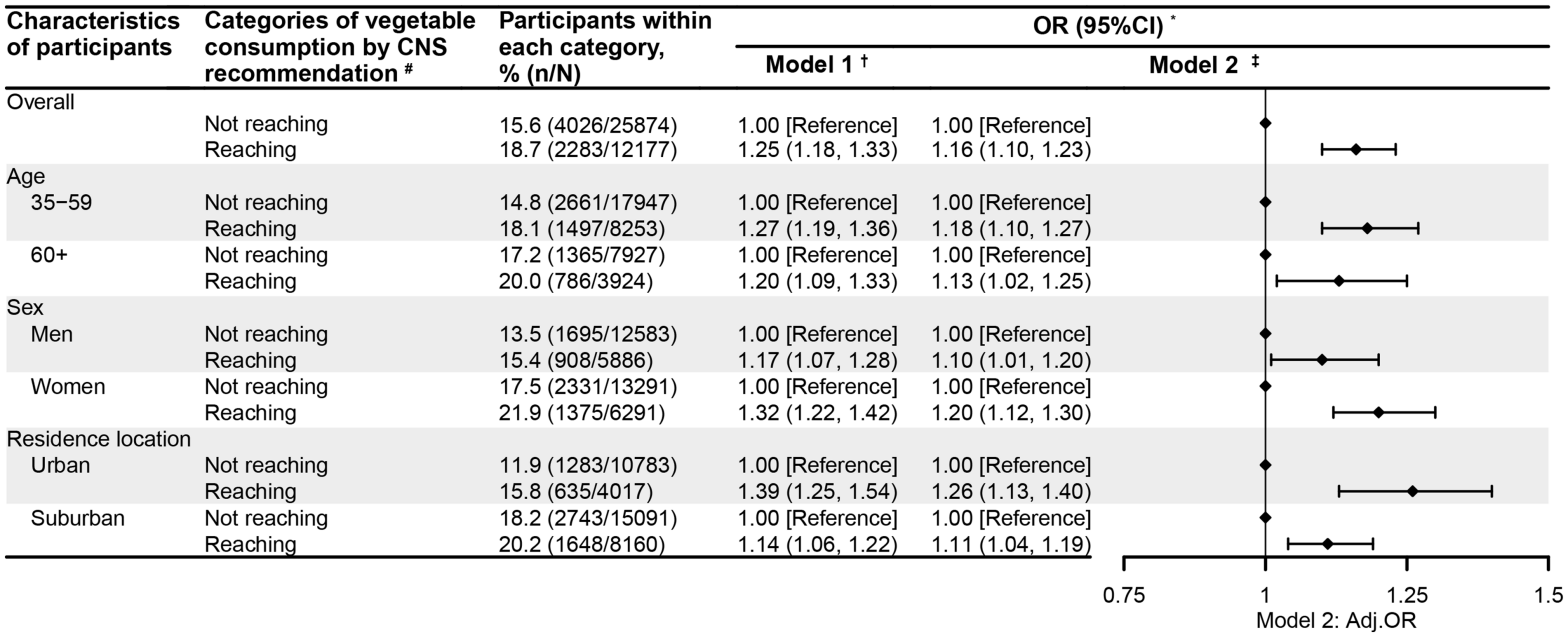
The relationship between the vegetable consumption recommendation and chronic gastric disorder among the participants aged 35 years and older in Nanjing municipality, China, 2023 (*N* = 38,051). * Gastric disorder refers to chronic gastritis or ulcer. † Model 1 was univariate logistic regression analysis, with the vegetable intake recommendation as the single predictor and community-level clustering effects controlled for. ‡ Model 2 was a multivariate logistic regression analysis, with adjustment for age (where applicable), sex (where applicable), residential location (where applicable), education, marital status, body weight status, smoking, drinking, physical activity, sedentary behavior, self-reported diabetes, self-reported hypertension, self-reported abnormal lipid profiles, family history of hypertension, family history of diabetes, meat intake, and community-level clustering effects in mixed logistic regression models. # CNS recommendation: consumption of ≥300 g/d of fresh vegetables was recommended for every Chinese adult by the Chinese Nutrition Society (CNS) in 2022.

**Figure 2:**
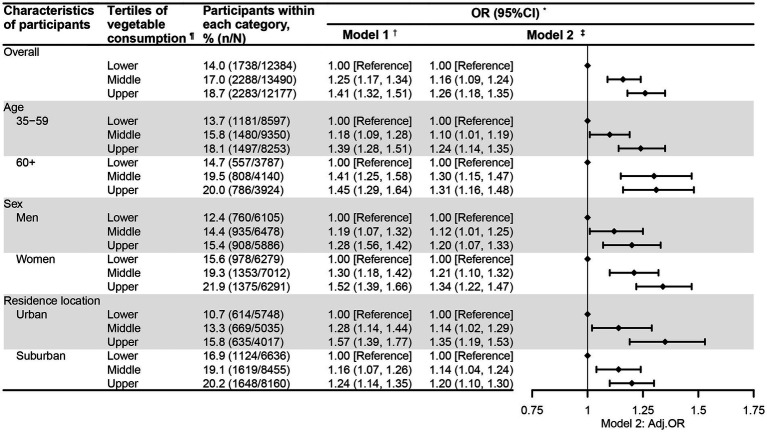
The relationship between vegetable consumption tertiles and chronic gastric disorder among the participants aged 35 years and older in Nanjing municipality, China, 2023 (*N* = 38,051). * Gastric disorder refers to chronic gastritis or ulcer. † Model 1 was univariate logistic regression analysis, with the vegetable intake tertile as the single predictor and community-level clustering effects controlled for. ‡ Model 2 was a multivariate logistic regression analysis, with adjustment for age (where applicable), sex (where applicable), residential location (where applicable), education, marital status, body weight status, smoking, drinking, physical activity, sedentary behavior, self-reported diabetes, self-reported hypertension, self-reported abnormal lipid profiles, family history of hypertension, family history of diabetes, meat intake, and community-level clustering effects in mixed logistic regression models. ¶ Vegetable consumption was classified into lower, middle, and upper tertiles, with cutoffs of 150 and 250 g/d.

Figures 1, 2 present the association between vegetable intake and CGD among the participants in this study. After adjustment for potential confounding factors, the participants whose vegetable intake met the recommendation had significantly higher odds of experiencing CGD (OR = 1.16, 95%CI = 1.10, 1.23) compared to those who did not meet the recommendation (Figure 1). Notably, this positive relationship between vegetable intake recommendation and CGD was consistently observed across the participants regardless of age, sex, or residential location. Moreover, when examining vegetable intake by tertiles, the adults in the high (OR = 1.26, 95%CI = 1.18, 1.35) and middle (OR = 1.16, 95%CI = 1.09, 1.24) tertiles were also more likely to experience CGD relative to those in the low tertile (Figure 2). Interestingly, the positive link between the tertiles of vegetable intake and CGD was also observed across participants regardless of age, sex, or residential location.

## Discussion

In this population study, we primarily aimed to investigate the relationship between vegetable intake and CGD among community-dwelling adults aged 35 years and older in regional China. It was found that: (1) the prevalence of self-reported CGD was 16.6% among all participants; (2) the participants who met the vegetable intake recommendation were more likely to experience CGD; (3) a positive link existed between vegetable intake tertiles and CGD; and (4) such positive associations between vegetable intake (measured either by the recommendation level or the intake tertile) and CGD were also observed across the participants regardless of age, sex, or residential location.

The positive relationship between vegetable intake and CGD observed in this study is not consistent with findings documented in existing literature (5–8). In previous studies, it has been reported that there is a negative (5, 6) or null relationship between vegetable intake and CGD (7, 8). Notably, the present study exhibited a completely different scenario—vegetable intake was positively associated with the risk of experiencing CGD both in the overall population and subgroups stratified by age, sex, or residential location. This highlights that the relationship between vegetable intake and CGD is really complex and deserves to be further investigated.

Regarding the two studies reporting a negative relationship between vegetable intake and CGD, both were cross-sectional surveys—one conducted in Japan and the other in China (5, 6). The Japanese study was conducted among 954 local residents aged 30 years or above in 1993 (5). Moreover, the independent variable was light-colored vegetable intake, which was measured by weekly consumption frequency (≥4 times/wk. *vs.* ≤3 times/wk), while the outcome variable was limited to CAG, which was determined based on serum levels of pepsinogen I (PG I), pepsinogen II (PGII), and the pepsinogen I/II ratio (PG I/II) (5). However, only age and sex were adjusted for in the analysis (5). On the other hand, the Chinese study was conducted among 6,863 Inner Mongolians aged 35 years and older in 2016–2017 (6). In this Chinese Inner Mongolian study, the independent variable was the combination of vegetable and fruit intake (categorized as “insufficient” or “sufficient” intake with the cutoff of 360 g/d and 180 g/d for vegetable and fruit intake, respectively), while the outcome variable was self-reported gastric precancerous lesions, including CAG, CGU, intestinal metaplasia, or dysplasia (6). In the analysis, age, sex, education, occupation, BMI, and family history of gastric cancer were considered (6).

As for the two cross-sectional studies documenting a null link between vegetable intake and CGD, interestingly, one was conducted in Japan and the other in China (7, 8). In the Japanese study reporting a null association, 1,017 community-dwelling adults aged 46 years and older from a town in Japan were analyzed (7). The independent variable, vegetable intake amount, was categorized into tertiles, while the outcome variable, CAG, was determined based on serum levels of PG I, PGII, and the ratio of PG I/II (7). Moreover, only age, sex, and smoking were controlled for in the analysis (7). On the other hand, the Chinese study reporting a null association recruited 574 outpatients, not community-dwelling residents, aged 18 years or above in Shandong province in 2018 (8). The independent variable was vegetable intake frequency, while the outcome variable was endoscopy-diagnosed CGD (8). In the analysis, covariates included BMI, education, residence, marital status, occupation, income, smoking, drinking, *Helicobacter pylori* (Hp) infection, history of diabetes, history of hypertension, history of anticoagulant use, and total energy intake (8).

Our study shared the same study design, a cross-sectional survey, with the four aforementioned studies (5–8). Notably, there were clear differences in terms of participant inclusion criteria, sample size, definitions of CGD, classifications of vegetable intake, and covariates between our study and the four investigations (5–8). Therefore, it is unlikely that a single explanation can account for the inconsistent findings between our study and the four previous surveys (5–8). To date, it has been well documented that vegetable intake is negatively associated with oxidative stress and inflammation (19, 20) but positively linked with antioxidant defense (20). In addition, vitamin C and *β*-carotene in vegetables may be able to maintain and improve lung function (21, 22). Unfortunately, these mechanisms may only explain the previously documented negative association between vegetable intake and CGD (5, 6) and are unlikely to explain the positive relationship examined in this study or the null associations reported in the two previous studies (7, 8).

However, several possible mechanisms may explain the findings observed in our study. First, it has been established that Hp infection is positively associated with CAG and CGU (23–27). Notably, Hp has been previously detected in fresh vegetables in different countries (23–25). In addition, more frequent consumption of vegetables (≥4 times/week), especially tomatoes and peppers, might increase the likelihood of Hp infection compared to lower vegetable intake (≤3 times/week) (26). Moreover, the positive relationship between vegetable intake and Hp infection was also reported among Chinese residents in an epidemiological study conducted in Shanghai, China (27). Second, dietary fibers in vegetables may be a driver of CGD (28, 29). Cellulose digestion largely depends on microbes, which require symbiotic microorganisms to ferment plant cell walls into volatile fatty acids (28). Typically, herbivores have foregut and/or hindgut fermentation systems that help break down cellulose and facilitate its easy digestion. In contrast, omnivores, including humans, do not have such a special digestive system (28). This implies that omnivores have very little fermentation capacity. Therefore, omnivores’ gastric mucosa has to perform increased mechanical activity to digest cellulose, which may lead to mucosal damage (28). Moreover, omnivores do not have endogenous cellulose enzymes to improve digestion (28). In addition, in some cases, cellulose sometimes can form phytobezoars and directly cause gastric ulcer (29).

This study has several strengths. First, more than 38,000 adults were randomly selected from community-dwelling residents. Moreover, they were representative of the overall population aged 35 years and older in a megacity. Second, the procedures used for data collection were standardized, and the instruments used to measure vegetable intake and key covariates were validated. Third, the recent recommendation was employed to assess the participants’ vegetable intake. Fourth, vegetable intake was not only dichotomized based on the CNS recommendation but also categorized into tertiles to evaluate its association with CGD. In addition, the vegetable intake–CGD relationship was examined across subgroups defined by age, sex, and residential location. Finally, an interesting but unexpected positive association between vegetable intake and CGD was observed consistently among participants overall and across subgroups stratified by age, sex, or residential location.

The limitations should also be acknowledged. First, due to the nature of the cross-sectional design, the positive link between vegetable intake and CGD may be partially explained by the fact that individuals with CGD consume more vegetables because they are more health-conscious than individuals without CGD. Therefore, no causal relationship between vegetable intake and CGD can be established in the study. Second, CGD was self-reported, which may have resulted in an underestimation of its prevalence. Third, only individuals with self-reported CGD were involved in the study. For these diagnosed patients, their lifestyle behaviors, including vegetable consumption, might have been intentionally modified. This might have introduced bias into the information collected on vegetable intake. Fourth, the outcome event was limited to CGA or CGU without differentiation between them. Fifth, an important risk factor of CGD, Hp infection, was not considered in the analysis due to a lack of data. Sixth, vegetable intake was measured based on the participants’ recall using the FFQ. Although the FFQ was validated, there might exist seasonal variations or long-term intake fluctuations. Seventh, due to a lack of data, we could not differentiate between types of vegetables. Consequently, we could not identify the specific vegetables that were associated with CGD in this study. Eighth, it has been documented that heavy metals, such as lead, mercury, and cadmium, are associated with CGDs (30, 31). Moreover, these heavy metals have been detected in both leafy and root vegetables in China (32, 33). Therefore, heavy metal contamination in vegetables might be a mediator between vegetable consumption and CGDs. Unfortunately, due to a lack of data on heavy metals in vegetables, we could not explore the role of heavy metal contamination as a potential mediator in the relationship between vegetable intake and CGDs in this study. Finally, the use of self-reported information for covariates also implies potential recall bias. Future well-designed longitudinal or experimental studies are encouraged to further investigate the relationship between vegetable intake and the risk of CGD worldwide.

In conclusion, the prevalence of self-reported CGD was as high as 16.6% among the community-dwelling adults aged 35 years and older in regional China. Vegetable intake was positively associated with CGD in the participants overall and subgroups stratified by age, sex, or residential location. This study suggests that vegetable intake may be an influencing factor for CGD among adults in China and indicates that population-level interventions targeting vegetable consumption might be an option for CGD prevention.

## Data Availability

The original contributions presented in the study are included in the article/supplementary material, further inquiries can be directed to the corresponding authors.
